# Anti-Cancer Effects of Green Tea Epigallocatchin-3-Gallate and Coffee Chlorogenic Acid

**DOI:** 10.3390/molecules25194553

**Published:** 2020-10-05

**Authors:** Sumio Hayakawa, Tomokazu Ohishi, Noriyuki Miyoshi, Yumiko Oishi, Yoriyuki Nakamura, Mamoru Isemura

**Affiliations:** 1 Department of Biochemistry and Molecular Biology, Graduate School of Medicine, Nippon Medical School, Bunkyo-ku, Tokyo 113-8602, Japan; y-oishi@nms.ac.jp; 2 Institute of Microbial Chemistry (BIKAKEN), Numazu, Microbial Chemistry Research Foundation, Shizuoka 410-0301, Japan; ohishit@bikaken.or.jp; 3 School of Nutritional and Environmental Sciences, University of Shizuoka, Suruga-ku, Shizuoka 422-8526, Japan; miyoshin@u-shizuoka-ken.ac.jp (N.M.); yori.naka222@u-shizuoka-ken.ac.jp (Y.N.)

**Keywords:** cancer, tea, coffee, EGCG, chlorogenic acid, ROS, AMPK, NF-κB

## Abstract

Tea and coffee are consumed worldwide and epidemiological and clinical studies have shown their health beneficial effects, including anti-cancer effects. Epigallocatechin gallate (EGCG) and chlorogenic acid (CGA) are the major components of green tea polyphenols and coffee polyphenols, respectively, and believed to be responsible for most of these effects. Although a large number of cell-based and animal experiments have provided convincing evidence to support the anti-cancer effects of green tea, coffee, EGCG, and CGA, human studies are still controversial and some studies have suggested even an increased risk for certain types of cancers such as esophageal and gynecological cancers with green tea consumption and bladder and lung cancers with coffee consumption. The reason for these inconsistent results may have been arisen from various confounding factors. Cell-based and animal studies have proposed several mechanisms whereby EGCG and CGA exert their anti-cancer effects. These components appear to share the common mechanisms, among which one related to reactive oxygen species is perhaps the most attractive. Meanwhile, EGCG and CGA have also different target molecules which might explain the site-specific differences of anti-cancer effects found in human studies. Further studies will be necessary to clarify what is the mechanism to cause such differences between green tea and coffee.

## 1. Introduction

**Figure 1:**
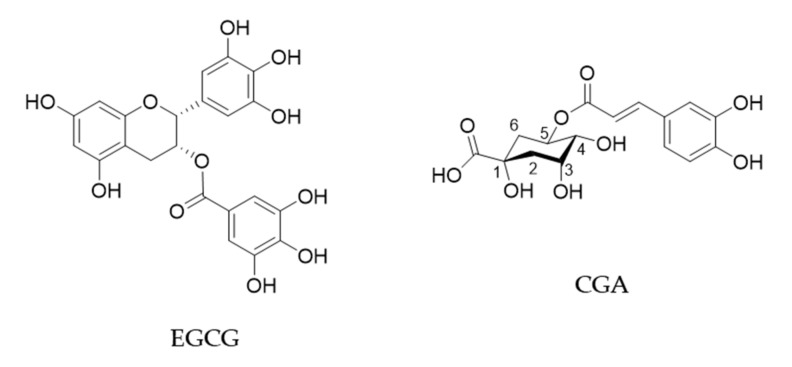
Chemical structures of EGCG and CGA.

Green tea is produced by processing of leaves of the plant *Camellia sinensis* (Theaceae) and is popularly consumed worldwide. Green tea has been shown to have beneficial effects on human health such as anti-cancer, anti-obesity, anti-diabetic, anti-cardiovascular, anti-infectious and anti-neurodegenerative effects [1,2]. (−)-Epigallocatechin gallate (EGCG) is the most abundant catechin in green tea and believed to be mostly responsible for these biological effects (Figure 1). A cup of green tea typically brewed from 2.5 g of tea leaves contains 240–320 mg of catechins, of which EGCG accounts for 60–65% [3].

Black tea is produced also from *C. sinensis* through enzymatic processing (so called fermentation) by intrinsic enzymes and microorganisms during which catechins can be polymerized to give catechin derivatives such as theaflavins and theasinensins [4]. Black tea has been shown to have physiological effects similar to those of green tea with lesser effects as compared with green tea due to its lower content of EGCG.

Coffee is also consumed worldwide and has various health effects. It contains about 2000 different chemicals and the major polyphenols are chlorogenic acid (CGA, Figure 1) and its derivatives which amount to about 3% *w*/*w* of roasted coffee powder [2,5]. A single cup of coffee may contain 20–675 mg of CGAs [6].

In this review, we discuss recent evidence from human studies to support the anti-cancer effects of consumption of green tea and coffee and mechanistic aspects of the actions of EGCG and CGA based on the results of cell-based and animal experiments. After the International Union of Pure and Applied Chemistry reversed the order of numbering of atoms on the quinic acid ring in 1976 and suggested the name 5-caffeoylquinic acid for chlorogenic acid instead of 3-caffeoylquinic acid [7,8], there has been some confusion in the nomenclature of chlorogenic acid. In this review, we use the term CGA according to the respective authors’ description. Caffeine is contained abundantly in tea and coffee and may contribute to the anti-cancer effects of these beverages. However, cell-based and animal studies have shown that EGCG as well as CGA exert anti-cancer effects by themselves as shown below. Therefore, for the safe of clarity, the current review focuses on EGCG and CGA but excludes any discussion on caffeine, which has already been comprehensively reviewed [9,10,11,12].

## 2. Anti-Cancer Effects of Green Tea

### 2.1. Human Studies on Green Tea

Several epidemiological studies have shown the anti-cancer effects of consumption of tea. A survey in 2013 conducted by Yang and Hong of prospective cohort and case controlled studies which had been reported by 2008 revealed that green tea consumption showed risk-reduction in a total of 39 cases of breast, colon, esophagus, kidney/bladder, lung, ovary, pancreas, prostate, stomach cancers, whereas 46 cases showed no risk-reduction [1,13]. In the case of black tea, 28 and 92 cases showed risk-reduction and no risk-reduction, respectively, for these cancers [13]. These findings suggest that green and black teas have a preventive effect in some types of cancer.

When observational epidemiological studies were reviewed on over 1,100,000 participants from 46 cohort studies and 85 case-control studies [14], in three studies involving 52,479 participants, a lower overall cancer incidence (summary relative risk (RR) = 0.83, 95% confidence interval (CI) = 0.65–1.07) was found for the highest intake of green tea compared with the lowest consumption. For most of the site-specific cancers, a decreased RR was found by this comparison. However, results were conflicting, since cohort studies in some cancer sites such as oesophageal, prostate and urinary tract cancer showed an increased RR. Table 1 added to show the effects of green tea on cancer, further explained in later text.

A recent review of 144 randomized controlled trials (RCTs) and case-control studies also provided evidence for beneficial effect of green tea in some cancer sites [14]. For example, the summary RR of prostate cancer in the green tea-supplemented participants was 0.50 (CI = 0.18–1.36) on the basis of three RCTs on 201 participants. However, the summary RR from 2 studies for gynecological cancer was 1.50 (CI = 0.41–5.48), indicating conflicting outcomes for some cancer sites.

In a recent survey of epidemiological studies reported from 2014 to 2018 on tea’s anti-cancer effects, Xu et al. [22] found that 5 and 2 studies of total 11 studies showed favorable and unfavorable effects of tea consumption, respectively, while 4 studies gave no effect, indicating a difficulty in drawing any conclusion.

More recent PubMed data search for human studies published from 2019 to April 2020 provided several papers showing anti-cancer effects of green tea [15,16,17,18,19,20,21] (Table 1). For example, in a population-based prospective cohort study in which 13,957 men and 16,374 women participated, the multiple-adjusted colon cancer RR (0.78, CI = 0.49–1.22) of men consuming ≥4 times of green tea daily was lower than that of the <1 time consumers, although no significant associations between green tea consumption and colorectal cancer (CRC) risk were found in men and women [18]. However, the same search revealed that 3 studies for cervical, liver and stomach cancers did not show significant risk reduction by green tea consumption [23,24,25]. Thus, human studies found health benefits of green tea consumption in many cases, but it is also true that there are several conflicting results probably due to incomplete elimination of confounding factors.

Polyphenon^®^ E is a standardized catechin preparation of green tea extract which was approved by the United States Food and Drug Administration in 2006 under the name of sinecatechins for the topical treatment of genital warts [26]. Its efficacy has been proven by several clinical studies as exemplified by a systematic review of three clinical trials in which Polyphenon^®^ E treatments resulted in significantly higher rates of complete clearance of baseline and new warts compared with controls with very low recurrence rates [27]. Genital warts are caused by human papilloma viruses (HPVs) such as types 6, 11 and 16 [28].

In view of successful application to various types of viral agents, Polyphenon^®^ E may be expected to be useful for the possible application to HPV-associated cancers such as cervical cancer and lymphocytic leukemia. A clinical trial in which 51 patients with HPV-infected cervical lesions were treated with Polyphenon^®^ E ointment or capsules or both, resulted in an overall 69% response rate as compared with that of 10% in untreated groups [29].

In a phase II trial on 42 patients with asymptomatic, chronic lymphocytic leukemia, it caused a sustained reduction of ≥20% of the absolute lymphocyte count in 31% of patients and ≥50% reduction in palpable lymphadenopathy in 69% patients [30]. Thus, future clinical intervention studies with Polyphenon^®^ E could lead to clear evidence for the anti-cancer effects of green tea.

### 2.2. Basic Research on Anti-Cancer Action of Green Tea and EGCG

A large number of cell-based and animal studies have provided evidence to support EGCG’s anti-cancer effects. For example, Wang et al. [31] demonstrated that EGCG decreased the numbers of intestinal aberrant crypt foci and colorectal tumors in rats treated with dimethylhydrazine. In a review article, Aggarwal et al. [32] summarized the results of 30 cell-based and 26 murine studies. Also, a comprehensive review by Gan et al. [33] summarized 63 cell-based studies reported in 2001–2015 and 21 animal studies reported in 2007–2015 which demonstrated the anti-cancer effects of EGCG. These authors suggested that these anti-cancer effects may be not due to EGCG itself but to its intracellular metabolites in view of EGCG’s low bioavailability.

These basic studies have also proposed mechanisms under which EGCG exerts these effects [1,2,3,4,5]. This review focuses mechanisms related to anti-oxidant and pro-oxidant effects, anti-inflammatory effects, anti-angiogenic effects, induction of apoptosis, modulation of epigenetic pathways and EGCG’s binding to cancer-related proteins which have been reviewed in many articles [26,34,35,36,37,38,39].

### 2.3. Mechanisms for Anti-Cancer Effects of EGCG

#### 2.3.1. Anti-Oxidant and Pro-Oxidant Effects

**Figure 2:**
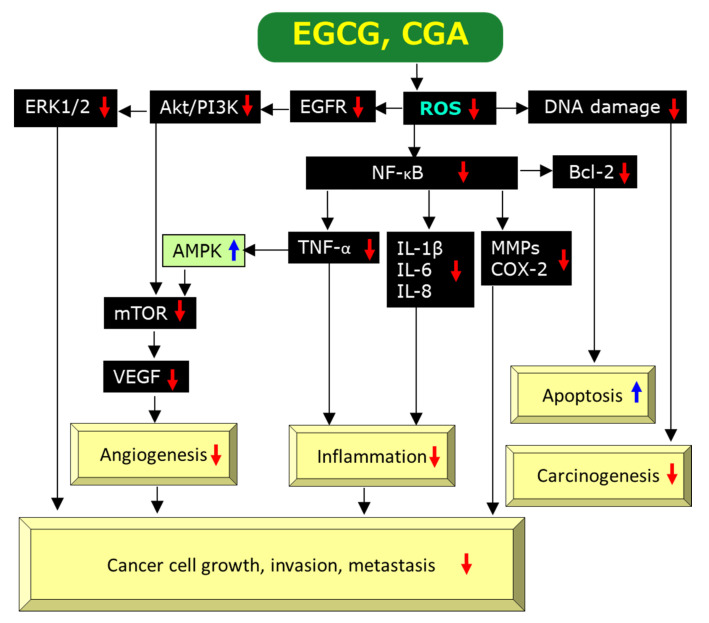
A possible mechanism by which EGCG and CGA exert anti-cancer effects via scavenging/downregulation of ROS. Red↓and blue↑marks represent downregulation/suppression and upregulation/stimulation, respectively.

EGCG is a prominent anti-oxidant and quenches reactive oxygen species (ROS), which facilitate oxidative DNA damage, mutagenesis, and tumor promotion, leading to anti-cancer effects [40]. EGCG can exhibit anti-oxidant activity through several mechanisms including catalytic metal chelation, hydrogen atom transfer, and electron transfer. Chemically, the anti-oxidant activity of EGCG can be interpreted by the existence of the polyhydroxyl structure and the gallate group which play key roles to scavenge free radicals and by the presence of phenolic groups with sensitivity to be oxidized, resulting in generation of a quinone [37,41]. Figure 2 illustrates a possible pathway through which EGCG exerts its anti-cancer actions via an anti-oxidant activity on the basis of present and previous findings and discussions [2,22,26,34,37,38,42,43,44]. Modulation of 5′-AMP activated protein kinase (AMPK) by tumor necrosis factor-α (TNF-α) is incorporated into Figure 2 based on the finding by Steinberg et al. [45] that TNF-α suppresses AMPK activity via transcriptional upregulation of protein phosphatase-2C, although this link remains to be explored in experiments using EGCG.

Actually, Bulboaca, et al. showed that i.p. administration of EGCG or liposomal EGCG improved the oxidative stress parameters such as malondialdehyde levels and nitric oxide (NO) synthesis as well as those of anti-oxidant status as evaluated by total anti-oxidant capacity and levels of thiols and catalase in plasma of rats treated with streptozotocin [46].

**Figure 3:**
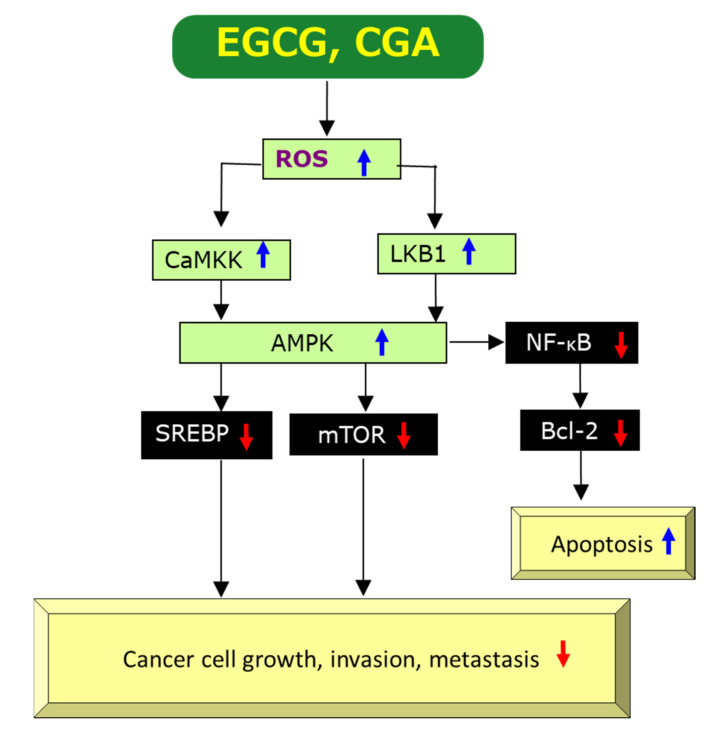
A possible mechanism by which EGCG and CGA exert anti-cancer effects via generation/upregulation of ROS. Red↓ and blue↑ marks represent downregulation/suppression and upregulation/stimulation, respectively.

Paradoxically, the pro-oxidant activity of EGCG has also been demonstrated by several studies and generation of ROS by EGCG is thought to be essential for the induction of apoptosis and inhibition of cell growth of cancer cells [37,40,42,47], as shown in Figure 3 which is compiled on the basis of previous data [2,34,42,48,49,50,51,52]. Since ROS generation induced by EGCG can upregulate AMPK, presumably through upregulation of Ca^2+^/calmodulin-dependent protein kinase kinase (CaMKK) and/or liver kinase B1 (LKB1) [49,50], leading to downregulation of mechanistic target of rapamycin kinase (mTOR) which results in anti-cancer effects. There are some reports to show downregulation of nuclear factor-κB (NF-κB) by AMPK, if not directly [51,52]. Xiang et al. [52] demonstrated that AMPK inhibited NF-κB activity using mice treated with complete Freund’s adjuvant. Therefore, ROS-mediated AMPK activation may also cause the downregulation of NF-κB, leading to anti-cancer effects through induction of apoptosis (Figure 3).

However, it is not clear at present by what mechanism EGCG act’s as an anti-oxidant or a pro-oxidant, although difference in cell types and different cellular concentrations including those of EGCG itself, metal ions, and the co-presence of other anti-oxidants may be important factors [42,53]. It can be assumed that either the anti-oxidant and pro-oxidant activities are involved in various mechanisms by which EGCG exerts anti-cancer effects (Figure 2 and Figure 3).

#### 2.3.2. Anti-Inflammatory Effects

Chronic inflammation is thought to have an important role on the onset and progression of human cancer by modulating the tumor microenvironment [54]. A number of studies have provided evidence EGCG’s anti-inflammatory effects. These studies found that EGCG can inhibit activation of transcription factors such as NF-κB, activating protein-1 (AP-1), MyD88-dependent signaling pathway, Toll-interleukin-1 receptor domain-containing adaptor inducing interferon-β (IFN-β)-dependent signaling pathways of Toll-like receptors, and expressions of inflammatory genes including cyclooxygenase (COX), NO synthase, and TNF-α [42,43,55]. Many of these actions may be interpreted by EGCG’s anti-oxidant activity (Figure 2). For example, ROS can induce NF-κB activation which in turn promotes biosynthesis of COX, NO, and TNF-α and, therefore, scavenging ROS by EGCG would lead to its anti-cancer effects [2,42] (Figure 2).

#### 2.3.3. Anti-Angiogenic Effects

Angiogenesis is the process characterized by the development of new blood vessels from the pre-existing vessels, which supply a tumor with oxygen and nutrients to allow optimal growth. Anti-angiogenesis is thought to be one of the most promising methods of cancer treatment [56].

Cancer cells can adopt to the hypoxic microenvironment by expressing hypoxia-inducible factors-1 (HIF-1) and thereby increasing the levels of its downstream target vascular endothelial growth factor (VEGF), which promotes tumor growth, angiogenesis, and metastasis [57,58,59]. EGCG was shown to decrease the protein expression of HIF-1α and VEGF proteins in gastric cancer SGC7901 cells under hypoxia induced by cobalt chloride [59].

In a study in which C57BL/6J mice inoculated with 10^6^ mouse breast cancer E0771 cells in the mammary gland fat pad, oral intake of EGCG at 50–100 mg/kg/day for 4 weeks reduced tumor weight, capillary density and tumor VEGF expression by 65, 30 and 23%, respectively, compared to control. EGCG at 50 μg/mL significantly inhibited the activation of HIF-1α and NF-κB as well as VEGF expression in cultured E0771 cells. These findings indicate that EGCG exerts anti-cancer effect by inhibiting angiogenesis mediated by the downregulation of VEGF, HIF-1α and NF-κB [60].

Wu et al. [61] found that EGCG inhibited the proliferation, viability, and cell cycle progression in three types of human thyroid carcinoma cells by decreasing the migration and invasion and increasing apoptosis. EGCG downregulated molecular signaling factors such as epidermal growth factor receptor (EGFR), extracellular signal-regulated kinase 1/2 (ERK1/2), and mitogen-activated protein kinase (MAPK) and inhibited tumor microvessel density dose-dependently in xenografts of these cells (Figure 2). Induction of angiogenesis by VEGF is caused by binding to its receptors on the surface of endothelial cells. Kondo et al. [62] reported that EGCG (1.56 to 100 μM) inhibited VEGF binding to its receptors in a dose-dependent manner.

Alternatively, EGCG’s anti-angiogenic action may be related to its pro-oxidant activity. EGCG may induce generation of ROS to promote apoptosis which is known to be the primary action of many anti-cancer drugs. ROS can up-regulate, perhaps indirectly, AMPK which modulates expressions of a number of proteins [3]. ROS-mediated activation of AMPK can lead to downregulation of mTOR, resulting in downregulation of VEGF (Figure 3) [2,36]. Therefore, EGCG’s pro-oxidant property can decrease the level of VEGF in cancer cells and tissues.

#### 2.3.4. Induction of Apoptosis

Induction of apoptosis or programmed cell death is one of the most important mechanisms for EGCG to exert anti-cancer effects. Several studies have provided evidence for the induction of apoptosis by EGCG and its mechanism of action. ROS can stimulate gene expression of B-cell lymphoma-2 (Bcl-2) via activation of NF-κB and therefore, EGCG’s scavenging activity of ROS is expected to downregulate the anti-apoptotic protein Bcl-2 (Figure 2), leading to apoptotic cell death of cancer cells (Figure 2).

Meanwhile, EGCG may induce apoptosis through enhancing ROS production (Figure 3). Das et al. [63] demonstrated that EGCG induced apoptosis via triggering ROS production with phosphorylation of p38 MAPK and activation of the redox-sensitive c-Jun N-terminal kinase-1 pathway. EGCG was also found to induce overexpression of apoptosis regulator Bcl-2 associated X (Bax) and activation of calpain, caspase-9, caspase-3, and caspase-8. It is noteworthy that EGCG did not induce apoptosis in human normal astrocytes [63].

Zan, et al. [64] reported that 5 and 20 μg/mL of EGCG induced apoptosis in breast cancer MCF-7 cells via the activation of caspase-9, caspase-3, and poly (ADP-ribose) polymerase-1 cleavage. Kwak et al. [65] also showed that 5 μg/mL of EGCG caused apoptosis in human cholangiocarcinoma HuCC-T1 cells through the increase of pro-apoptotic protein Bax and activation of caspase-9 and caspase-3, and cytochrome c release. Similarly, Jian et al. [66] found that EGCG induced apoptosis in human hepatocellular carcinoma (HCC) HepG2 cells and rat pheochromocytoma PC12 cells through downregulation of Bcl-2 and upregulation of Bax.

Sterol-response element binding protein-1 (SREBP-1), a nuclear transcription factor mainly involved in lipid metabolism, is also downregulated by AMPK (Figure 3). SREBP-1 is expressed at higher levels in patients with large tumor size, high histological grade and advanced tumor-node-metastasis stages. Downregulation of SREBP-1 inhibited cell proliferation and induced apoptosis in both HepG2 and MHCC97L cells and SREBP-1 knockdown inhibited cell migration and invasion in both cancer cell types [67]. Since EGCG’s suppression of the expression of SREBP-1 through the activation of the AMPK pathway in sebocytes was reported [68], this EGCG’s inhibition may be expected to contribute to its anti-cancer effect (Figure 3).

#### 2.3.5. Epigenetic Modifications

Epigenetic modifications represent post-translational changes in histones and DNA such as methylation and acetylation as well as dysregulation of microRNAs (miRNAs) expression [69]. Noncoding RNAs (ncRNAs) consist of miRNAs and long noncoding RNAs (lncRNAs) where miRNAs are defined as small single-stranded molecules (ca. 20 to 25 nucleotides) and lncRNAs as RNA molecules larger than 200 nucleotides. These ncRNAs are implicated in various cellular processes through regulating gene expression at the transcriptional and post-transcriptional level and thought to play roles in various diseases including cancer [70].

One mechanism involved in anti-cancer effects exerted by EGCG is such epigenetic modifications. The inhibitors of DNA methyltransferase (DNMT) and histone deacetylase (HDAC) are expected to be promising anti-cancer drugs. Fang et al. [71] demonstrated that EGCG inhibited DNMT activity with a Ki of 6.89 μM. Similarly, Pal et al. [72] showed that 10 μg/mL of EGCG decreased the proliferation of HeLa cells and expression of DNMT-1. Khan et al. [73] showed that EGCG inhibited the expression of DNMT-3B and HDAC-1 in a time-dependent manner in human cervical carcinoma HeLa cells.

In a review by Aggarwal et al. [32] the authors summarized the effects of EGCG on various cancers reported in 11 studies. In an experiment using cervical carcinoma cell lines, EGCG inhibited HeLa cells growth in a dose- and time-dependent manner [74]. EGCG caused downregulation of miR-125b and upregulation of miR-210 and miR-29 in HeLa cells and also upregulation of miR-210 and miR-29 expressions in CaSKi and SiHa cells. EGCG’s upregulation of miR-210 was also found in experiments using lung cancer cells and a nude mouse model [75]. Overexpression of miR-210 led to reduction in cell proliferation and anchorage-independent cell growth [75].

In addition, Aggarwal et al. [32] described three studies in which EGCG upregulated the let-7 family miRNAs, which were implicated to function as a tumor suppressor and cause down-regulation of high mobility group-A2, a target gene related to tumor progression via 67-kDa laminin receptor (67LR)-binding in melanoma cells [76].

In another study, EGCG was demonstrated to decrease the expression of p53 gene-targeting miRNAs (miR-25, miR-92, miR-141, and miR-200a) in multiple myeloma cells [77]. The data suggest that EGCG can reverse the elevated expression of miRNAs which downregulate p53 in cancer cells and exert its anti-cancer effect via recovery of the activity of tumor suppressor p53. In harmony with this finding, EGCG was shown to stabilize p53 to upregulate its transcriptional activity leading to apoptosis in prostate cancer LNCaP cells [78]. It should be noted that EGCG downregulated miR-25 and miR-92 in multiple myeloma cells but upregulated them in HCC [77]. The difference may be due to cell-specific effect but further studies are required to understand the EGCG’s effects on miRNAs.

Hu et al. [79] demonstrated that EGCG inhibited the growth of lung cancer A549 and NCI-H460 cells in a concentration-dependent manner. They identified an upregulation of RP1-74M1.3, AC087273.2, SNAI3-AS1, LINC02532, and AC007319.1 lncRNAs and downregulation of HMMR-AS1, AL392089.1, PSMC3IP, LINC02643, and H19 lncRNAs in EGCG-treated A549 cells. These lncRNAs are distributed across nearly all human chromosomes and EGCG affected lncRNAs expressions, suggesting that EGCG can regulate the expression of ncRNAs to exert anti-cancer activity in several types of cancer.

#### 2.3.6. Molecular Docking Analysis of EGCG’s Binding to Cancer-Related Proteins

A number of studies have demonstrated that the binding affinity of EGCG to proteins contributes to its anti-cancer mechanism. There are several physicochemical methods to examine molecular interaction between EGCG and proteins. In 1997, our research group conducted for the first time affinity chromatography using EGCG-agarose gel to demonstrate that EGCG binds to matrix metalloproteinase (MMP)-2 and MMP-9 which are intimately associated with cancer cell invasion and metastasis [80]. EGCG inhibited activities of these enzymes, leading to anti-cancer effects like batimastat (BB-94), a synthetic MMP inhibitor that inhibits tumor growth, local invasion, and lung metastasis of orthotopic metastatic human HCC in nude mice model [80,81]. Later, the binding interaction between EGCG and MMP-2 and MMP-9 was confirmed by computational molecular docking analysis (MDA) [82].

In our previous review article, we discussed binding interactions between EGCG and other cancer-related proteins revealed by affinity chromatography and pull-down methods using EGCG-agarose gel [82]. These include fibronectin, vimentin, heat shock protein 90, glucose-regulated protein 78 kDa (GRP78), insulin-like growth factor-1 receptor, Src-related proto-oncogene Fyn protein, ζ chain-associated 70-kDa protein, Ras-GTPase-activating protein Src homology (SH3) domain-binding protein-1, peptidyl-prolyl cis-transisomerase, and TNF receptor-associated factor-6. Most of these interactions were confirmed by MDA [82].

Similarly MDA revealed the binding interaction between EGCG and VEGF, VEGF receptors, tyrosine kinases, urokinase, chymotrypsin, DNMT, protein phosphatases, and signal transducer and activator of transcription-3 [83]. These protein-binding interactions are likely to be involved in EGCG’s anti-cancer effects.

#### 2.3.7. Roles of 67LR in EGCG’s Anti-Cancer Effects

One of the EGCG’s most important interactions may be that with 67LR, which was discovered by a surface plasmon resonance technique as discussed in several papers [84,85,86]. EGCG was shown to bind 67LR at physiologically available concentrations (0.1–1.0 μM) and to mediate many of its beneficial activities, including anti-cancer effect. EGCG binding to 67LR via eukaryotic elongation factor-1A causes the phosphorylation of myosin phosphatase targeting subunit-1 and activates myosin phosphatase which dephosphorylates its substrates such as myosin regulatory light chain, resulting in actin cytoskeleton rearrangement leading to cell growth inhibition [84,86].

## 3. Anti-Cancer Effects of Coffee

### 3.1. Human Studies on Anti-Cancer Effects of Coffee

Coffee is the second most consumed beverage worldwide after tea. Some early epidemiological studies suggested that coffee consumption was associated with an increased cancer risk [87]. For example, Yu et al. [88] described that daily coffee consumption is a risk factor in females for renal cell carcinoma. Based on the results of 32 epidemiological studies, Wierzejska found that several studies showed that coffee consumption had no or even unfavorable association with colorectal, breast, bladder, prostate, lung and pancreatic cancers, but emphasized that other studies showed promising results for these cancers and liver cancer [87].

Several early RCT suggested the coffee’s favorable effects on cancers as exemplified by following findings: When 64 participants were randomly assigned into two groups and consumed 1000 mL of cafetière coffee daily or no coffee for intervention and washout periods, the result indicated that unfiltered coffee significantly increased the glutathione content by 8% in the colorectal mucosa and by 15% in plasma [89]. The increase in the detoxification capacity and anti-mutagenic properties in the colorectal mucosa through an increase in glutathione concentration suggests the possible lowering effect on the colon cancer risk [89].

A clinical trial with 10 participants found that consumption of 1L unfiltered coffee/day over 5 days resulted in a weak induction of glutathione-*S*-transferases (GSTs) and 3-fold increase in induction of placental type GST in blood, although other clinical markers for organ damage such as creatinine, aminotransferases, and alkaline phosphatase were not altered [90]. The finding suggests that coffee’s induction of placental type GST may lead to protection from chemical carcinogenesis.

In a controlled intervention trial with a cross-over design with 38 participants, consumption of 800 mL coffee daily over 5 days demonstrated the decrease by 12.3% in the extent of DNA-migration attributable to formation of oxidized purines, although other biochemical parameters such as the total anti-oxidant levels in plasma, glutathione concentrations in blood, and the activities of superoxide dismutase and glutathione peroxidase in lymphocytes were not markedly altered. The result indicates that coffee consumption prevents endogenous formation of oxidative DNA-damage in human [91].

Recent evidence has also suggested that coffee drinking may have health benefits on some types of cancer. A review by an International Agency for Research on Cancer working group conducted in 2016 on a large number of epidemiological and experimental studies on anti-cancer effects of coffee found an inverse association for liver and endometrial cancers [92].

Similarly, a comprehensive review of the beneficial effects of coffee and its components on gastrointestinal and liver carcinogenesis summarized observational epidemiological studies: four studies on oropharyngeal cancer, four on esophagus cancer, four on stomach cancer, four on CRC, and seven HCC [11]. Comparing the highest and lowest consumptions, all study results showed 31–37% risk reduction in oropharyngeal cancer, no risk reduction in esophagus cancer, no risk reduction in CRC and 34–43% risk reduction in HCC, although some subgroup analyses gave different results. In the case of stomach cancer, one study found reduced risk, two no effect and one increased risk. These results indicate that the coffee’s benefit might be limited to liver cancer.

In addition, a recent meta-analysis of observational studies on associations between coffee intake and 26 different cancers including 364,749 cancer cases provided evidence to show that coffee intake is inversely associated with endometrial cancer, liver cancer, melanoma, oral cancer, and oral/pharyngeal cancer [93]. Additional evidence was also obtained to suggest the reduced risk of cancers of the mouth, pharynx and larynx, and skin cancer. Coffee consumption may also be inversely associated with breast, colon, colorectal, esophageal and nonmelanoma skin cancers.

Conversely, the same analysis showed the conflicting result whereby a higher coffee intake was associated with an increased risk of childhood acute lymphocytic leukemia, bladder cancer, and possibly lung cancer [93]. Similarly, a more recent pooled analysis of 12 cohort studies, comprising of 2601 cases out of 501,604 participants found a significantly increased risk for bladder cancer in male smokers: when compared the consumers of >4 cups/day with the non-consumers, hazard ratios were 1.75 (CI = 1.27–2.42) for current smokers and 1.44 (CI = 1.12–1.85) for former smokers [94].

In a review on the association of CRC risk with coffee, caffeinated coffee and decaffeinated consumptions, Buldak et al. [10] discussed eight, seven and three observational epidemiological studies showing no association, inverse association, and association with increased risk, respectively. These authors pointed out that caffeine is not an important component for coffee to exhibit the anti-cancer activity, since several studies found significant inverse correlation for both caffeinated and decaffeinated coffee consumptions.

A recent RCT on 160 healthy human subjects who consumed 3 or 5 cups of coffee per day for 8 weeks found that blood pressure, oxidation of DNA and lipids, blood levels of glucose, insulin, cholesterol, triglycerides, and inflammatory markers were unchanged, although a slight elevation of serum creatinine level and a significant elevation of serum γ-glutamyltransaminase levels in the 5 cups/day group [95]. The results indicated no detectable effects, either beneficial or harmful, on human health.

Thus, these findings from clinical studies are conflicting. The recall bias and confounding effects including tobacco smoking, a method for brewing coffee, differences in ingredients, and genetic background may contribute to these differences.

### 3.2. Comparison of Anti-Cancer Effects of Tea and Coffee in Simultaneous Human Studies

Several epidemiological studies have examined the anti-cancer effects of tea and coffee at the same time. For example, the European Prospective Investigation into Cancer and Nutrition on 486,799 subjects with a median follow-up of 11 years found that both coffee and tea intakes were inversely associated with HCC risk. Coffee and tea consumers in the highest compared to the lowest quintile had lower HCC risk by 72% and 59%, respectively [96]. In a study in which 10,399 of total 97,334 subjects developed cancers of 145 head and neck, 99 oesophageal, 136 stomach, 1137 lung, 1703 breast, 257 endometrial, 162 ovarian, 3037 prostate, 318 kidney, 398 bladder, 103 gliomas, and 106 thyroid, tea consumption of ≥1 cups/day was inversely associated with cancer overall combined (RR = 0.95, CI = 0.94–0.96) as compared to <1 cup consumption, but no association of coffee consumption with the risk of all cancers combined was found. However, coffee intake decreased a risk for endometrial cancer (RR = 0.69, 95% CI = 0.52–0.91 for ≥2 cups per day), while tea did not [97].

A meta-analysis of 12 case-control studies, comprising a total of 3649 cases and 5705 controls found that a high maternal coffee consumption increased a risk of acute lymphoblastic leukemia in childhood (OR = 1.43), whereas low to moderate tea consumption was inversely associated (odds ratio (OR) = 0.85, CI = 0.75–0.97), although the trend was not significant [98].

Table 2 shows a brief comparison of anti-cancer effects of tea and coffee in simultaneous studies reported since 2018 based on the Medline data base. Several investigations revealed that tea and coffee may have different effects in some cancer types. It is noticeable that coffee may increase a risk in certain types of cancer (bladder cancer, lung cancer, and childhood leukemia) in line with the finding from aforementioned studies which examined effects of either tea or coffee, individually [93].

The reason for the difference is not known at present. As pointed out by Milne et al. [99], the fact that both tea and coffee contain numerous different compounds, are prepared by various methods, and have differences in bioavailability makes it difficult to determine the factor(s) involved in the difference.

### 3.3. Basic Research on Anti-Cancer Action of Coffee and CGA

A number of cell-based and animal studies have provided evidence to support anti-cancer effects of coffee and CGA [117,118,119,120]. Salomone et al. [118] have elegantly discussed molecular basis of anti-cancer effects of coffee and some of its components including CGA. They summarized the results of 10 animal studies showing anti-cancer effects of coffee and CGA as examined in experimental models of liver cancer. For example, in an experiment of Miura et al. [119] coffee inhibited the proliferation and invasion of rat ascites hepatoma AH109A cells and the serum from rats given coffee orally also exhibited anti-proliferative and anti-invasive activities against these cells.

Similarly, Buldak et al. [10] reviewed the human and basic studies on anti-cancer effects of coffee and its components on CRC. These authors discussed the results of three cell-based studies on CGA. In an experiment by Hou et al. [120], CGA was shown to inhibit the proliferation of human colon cancer HCT116 and HT29 cells. CGA induced ROS generation and cell cycle arrest at the S phase, and suppressed the activation of ERK in both cell types, leading to the anti-cancer effect against CRC.

More recently, Romualdo et al. [11] discussed these issues on the basis of animal studies of the effects of coffee and CGA on oral and esophagus cancers (four studies), colon cancer (nine studies) and HCC (four studies). For example, the included data showed that two of four studies of coffee and four of five studies of CGA demonstrated beneficial effects on colon cancer. These authors summarized the mechanistic aspects of CGA’s action which are associated with molecular pathways involving ROS and others such as Bax, interleukin (IL)-8, caspase-3, MMPs and miR-21. Although these articles also reviewed comprehensively other coffee components such as caffeine, caffeic acid, and kahweol, this review focuses CGA which is considered to be the major player in the coffee’s anti-cancer mechanism as discuss below.

### 3.4. Mechanisms of CGA’s Action against Cancer

#### 3.4.1. Anti-Oxidant and Pro-Oxidant Properties, Anti-Inflammatory Effects, Anti-Angiogenic Effects and Apoptosis-Inducing Activity of CGA

CGA’s involvement in anti- and pro-oxidant actions, anti-inflammatory effects, anti-angiogenic effects, and apoptosis-inducing activity of coffee has often been documented [7,11,48,118,120,121,122]. Examples are as follows:

Cha et al. [123] demonstrated that UVB gave damage to cellular DNA in human HaCaT cells, as demonstrated in a comet assay, but CGA pre-treatment prior to UVB irradiation prevented oxidative DNA damage and increased cell viability. Rakshit et al. [124] found that CGA induced an early accumulation of intracellular ROS and apoptosis in chronic myeloid leukemia cells.

Feng et al. [121] found that CGA inhibited the proliferation of A549 human cancer cells in vitro and that CGA suppressed 12-*O*-tetradecanoylphorbol-13-acetate (TPA)-induced neoplastic transformation of JB6 P+ cells. CGA decreased UVB- or TPA-induced transactivation of AP-1 and NF-κB, the phosphorylation of c-Jun NH_2_-terminal kinases, p38 kinase, and MAPK kinase-4 induced by UVB or TPA. The results also showed that CGA stimulated the nuclear translocation of NF-E2-related factor (Nrf2) as well as subsequent induction of GST-A1 anti-oxidant response element (ARE)-mediated GST activity. These results suggest that the chemopreventive effects of CGA may be through its up-regulation of cellular anti-oxidant enzymes via stimulation of Nrf2 and suppression of ROS-mediated NF-κB, AP-1, and MAPK activation [121].

Liang and Kitts reviewed anti-oxidative and anti-inflammatory effects of CGA [122]. They discussed five cell-based studies and two animal experiments, in which downregulation of ROS was demonstrated, and 10 experiments, most of which showed downregulation of inflammation-related cytokines such as IL-1β, TNF-α and IL-6. Four such studies revealed downregulation of NF-κB.

When anti-inflammatory effect of CGA was examined in lipopolysaccharide-stimulated murine RAW 264.7 macrophages and BV2 microglial cells, CGA inhibited NO production, the expression of COX-2 and inducible NO synthase, and attenuated pro-inflammatory cytokines such as IL-1β and TNF-α via inhibition of the nuclear translocation of NF-κB [125].

In an attempt to evaluate the effects of CGA on retinal neovascularization in a mouse model of oxygen-induced retinopathy, Kim et al. [126] found that CGA inhibited VEGF-mediated tube formation in human vascular endothelial cells and that the neovascular area was significantly smaller in CGA-treated mice than in the vehicle-treated mice, demonstrating the CGA’s anti-angiogenic property.

Most of these results are related to modulation of ROS and the aforementioned mechanisms by which EGCG exerts its anti-cancer effects might be applicable to CGA. Figure 2 and Figure 3 show possible modulations by CGA, although individual pathways in which CGA is involved have not necessarily been reported. Figure 2 illustrates CGA’s downregulation of ROS [2,11,48,120,127], DNA damage [123], EGFR [127], Akt/phosphatidyl 3-inositol kinase (PI3K) [127], ERK1/2 [11,127], NF-κB [2,121,125,127,128], TNF-α [2,122], IL-1β [122], IL-8 [2,11], IL-6 [122], MMPs [2,11], COX-2 [125,128], Bcl-2 [10,129], mTOR [10,127], VEGF [126], and upregulation of AMPK [7,49]. Although CGA’s upregulation of CaMKK and LKB1 shown in Figure 3 has not been determined yet, Park et al. [130] reported upregulation of them by neochlorogenic acid, an isomer of CGA. Thus, EGCG and CGA would be expected to exert anti-cancer effects by modulating similar molecular pathways to each other in many cases.

#### 3.4.2. Epigenetic Modification by CGA

Increased levels or alterations in the function of DNMT-1 are associated with the inactivation of tumor suppressor genes. Liu et al. [131] showed that CGA inhibited the proliferation, colony formation, invasion, and metastasis of HepG2 cells both in vitro and in vivo by down-regulating DNMT-1 protein expression, which enhanced p53 and p21 activity, and resulting in a significant reduction in cell proliferation and metastasis. Moreover, CGA inactivated ERK1/2 and reduced MMP-2 and MMP-9 expression in HepG2 cells. These findings suggest that CGA exhibits anti-cancer effects through several pathways. Using synthetic DNA substrates, Lee and Zhu found that CGA inhibited human DNMT-1 activity with an IC_50_ value of 0.9 μM. In MCF-7 and MAD-MB-231 human breast cancer cells, CGA inhibited the methylation of the promoter region of the retinoic acid receptor β gene [132].

Mira and Shimizu found the methanol extract of the medical herb *Angelica shikokiana* and some of its components including CGA showed cytotoxicity against various cultured cells and inhibited tubulin polymerization [133]. CGA was shown to inhibit activity of HDAC-8 (IC_50_ = 8.62 μM).

Hongtao et al. [134] found that CGA blocked the proliferation of non-small cell lung cancer cells. CGA inhibited HDAC-6 and MMP-2 activities through reduction in expression of acetylated NF-κB, the level of which is positively associated with the transcription of pro-inflammatory cytokines [134]. The results suggest CGA’s anti-cancer effect through suppression of HDAC-6 activity. In line with these findings, an inhibition experiment with HeLa cell nuclear extracts and MDA conducted by Bora-Tatar et al. [135] demonstrated that CGA is the highly potent inhibitor compared to sodium butyrate, which is a well-known HDAC inhibitor.

Several studies have examined effects of CGA on miRNAs. The results of a study, in which hepatic stellate LX2 cells and CCl_4_-induced liver fibrosis model rats are used, indicated that CGA inhibited the mRNA expression of miR-21, Smad7, connective tissue growth factor, α-smooth muscle actin, tissue inhibitor of metalloproteinase 1, MMP-9, and transforming growth factor-β1 (TGF-β1), suggesting that CGA relieves liver fibrosis through the miR-21-regulated TGF-β1/Smad7 signaling pathway [136]. Similar results were reported by Wang et al. [137] who showed that CGA could inhibit schistosomiasis-induced liver fibrosis through IL-13/miR-21/Smad7 signaling interactions in LX2 cells and schistosome-infected mice. Since liver fibrosis is a key factor for the risk of HCC [138], CGA might be useful for its prevention.

Induction of cancer differentiation may be a promising strategy to treat cancer. CGA reduced proliferation rate and migration/invasion ability in human hepatoma Huh7 and lung H446 cancer cells through elevation of small ubiquitin like modifier-1 expression by acting on its 3′-untranslated region and stabilizing the mRNA, leading to downregulation of miR-17 family member miR-20a, -93, and -106b. The xenograft experiments using these cells gave similar results. NOD/SCID mice which received i.p. administration of 25–200 mg/kg/day of CGA demonstrated tumor growth inhibition and administration of 25 mg/kg caused downregulation of miR-17 family members [139].

#### 3.4.3. MDA of CGA’s Binding to Cancer-Related Proteins

The results of MDA showed that quercetin, rutin, and CGA can bind to MMP-1, MMP-3, and MMP-10 [140]. MDA of CGA and carbonic anhydrase IX showed the high affinity which is attributable to the strong interaction with enzyme active site through the formation of eight hydrogen bonds with the active site residue [141].

MDA for natural products which may interfere with SARS-CoV-2 attachment to the host cell found that CGA had the good average binding affinity to the cell-surface heat shock protein A5 (GRP78) of −7.10  ±  0.96 kcal/mol [142].

P-glycoprotein is associated with multidrug resistance as a drug efflux protein. CGA exhibited anti-proliferative effect on the mouse T-cell lymphoma L5178 cells with an IC_50_ = 0.06 ± 0.003 μg/mL and reversed multidrug resistance. MDA revealed that CGA can bind to three different sites which are known to be bound by verapamil with similar binding energies of around 7 kcal/mol [143].

CGA induced apoptosis in a dose-dependent manner with an IC_50_ of 75.88 ± 4.54 μg/mL and 52.5 ± 4.72 μg/mL in MDAMB-231 and MCF-7 cells, respectively. CGA binds to protein kinase C in a concentration-dependent manner with a Kd of 28.84 ± 3.95 μM and MDA suggested that CGA fits into the C1b domain of protein kinase C [144].

By UPLC-MS/MS analysis, Taha et al. [145] identified 22 compounds in the extracts of the fruits of *Nandina domestica* Thunb. which have served as a folk medicine in therapies of some types of cancer. MDA of CGA and some other compounds revealed strong interactions with the cancer-related proteins Akt1, caspase-3, MAPK-1 and tumor suppressor TP53.

## 4. Conclusions

The present review has discussed the anti-cancer effects of green tea and coffee based on epidemiological and intervention studies. These studies have provided evidence to show favorable effects on some types of cancer such as breast, colon, lung and blood cancers by green tea consumption (Table 1 and Table 2) and those such as liver, endometrial, and skin cancers by coffee consumption (Table 2). Thus, green tea and coffee are likely to have some differences in site-specific anti-cancer effects.

Meanwhile, considerable studies have reported conflicting results, presumably due to confounding factors such as the method of quantifying consumption, beverage temperature, cigarette smoking, alcohol consumption, and differences in genetic and environmental factors such as race, sex, and age, lifestyle, intestinal microbiota and genetic polymorphisms [2,34,42]. Therefore, more rigorous human studies are necessary to establish the anti-cancer effects of consumption of these beverages.

This review has also provided evidence to show anti-cancer effects of EGCG and CGA based on cellular and animal experiments. These experiments have proposed several mechanisms through which EGCG and CGA exert their anti-cancer effects. Among them, the mechanism involving downregulation of ROS appears to explain commonly their anti-cancer actions (Figure 2). Another important mechanism may be related to ROS generation as shown in Figure 3.

Meanwhile, interpretations for the different anti-cancer effects between green tea and coffee need to be clarified. One possible explanation is the difference in interaction with target molecules. For example, binding interaction has not been reported between CGA and 67LR that is an important target of EGCG. The difference in co-existing molecules may also contribute to the different effects. For example, animal experiments showed that caffeic acid present in coffee exhibited carcinogenicity in the rat stomach [146,147] which may cancel the CGA’s anti-cancer effect. Differences in by-products such as acrylamide generated during roasting and brewing and heavy metals and aflatoxin A which may have contaminated can be a reason [11,22,89].

In addition, some studies suggested a risk increase in certain types of cancers such as esophageal and gynecological cancers in green tea consumption [14] and bladder and lung cancers in coffee consumption (Table 2). The reason for these observations may be clarified in future studies.

## Figures and Tables

**Table 1:** Recent observational epidemiological studies on anti-cancer effects of green tea.

Cancer Type	Evaluation: Decrease (↓) or No Effect (+/−) in Cancer Risk	Hazard Risk (HR) or Odds Ratio (OR) or Relative Risk (RR) [Confidence Interval]	Note	Reference
Breast cancer	↓	HR = 0.82 [0.70–0.95] for ≥5 vs. 0 cups/day	Cohort study onwomen with family history of breast cancer	[15]
Breast cancer	↓	HR = 0.86 [0.75–0.99] for highest vs. lowest intake	Meta-analysis of 16 cohort and case-control studies	[16]
Breast cancer	↓	OR = 0.83 [0.72–0.96]	Meta-analysis of 14 case-control studies	[17]
Colorectal cancer	+/−		Cohort study on men and women	[18]
Colon cancer	↓	RR = 1.32 [0.90–1.94] for once/day vs. less than once/day RR = 0.76 [0.57–1.02] for 2–3 times/day RR = 0.78 [0.49–1.22] for ≥4 times/day	Cohort study on men	[18]
Head and neck squamous cell carcinoma	↓	OR = 0.29 [0.16–0.52] for <1 cup/day vs. no intake OR = 0.38 [0.17–0.86] for ≥1 cup/day vs. no intake	Case-control studyon men and women	[19]
Hematologic neoplasms	↓	HR = 0.65 [0.42–1.00] for ≤2 cups/day vs. no intake HR = 0.73 [0.47–1.13] for 3–4 cups/day vs. no intake HR = 0.63 [0.42–0.96] for ≥5 cups/day vs. no intake	Cohort studyon men and women	[20]
Total cancer	↓	HR = 0.89 [0.83–0.96] for 1–2 cups/day vs. <1 cup/day HR = 0.91 [0.85–0.98], for 3–4 cups/day vs. <1 cup/day	Meta-analysis on 8 cohort study on women	[21]

**Table 2:** Comparison of anticancer effects in humans between tea and coffee.

Cancer Type	Tea/Green Tea/Black Tea *	Coffee/Caffeinated Coffee/Decaffeinated Coffee *	Type of Epidemiological Study [Reference]
Bladder	↓	+/−	Cohort study [100]
Bladder	+/−	↑	Meta-analysis of cohort study and case-control study [101]
Brain	↓	↓	Meta-analysis of cohort study and case-control study [102]
Breast	+/−	+/−	Cohort study [103]
Colorectal	+/−	+/−	Cohort study [104]
Colorectal	↓	+/−	Case-control study [105]
Endometrial	+/−	↓	Case-control study [103]
Glioma	↓	+/−	Cohort study [106]
Glioma	↓	↓	Case-control study [107]
Leukemia, acute myeloid	+/−	+/−	Cohort study [108]
Leukemia, childhood acute myeloid	+/−	↑	Meta-analysis of case-control study [109]
Leukemia, childhood acute lymphoblastic	+/−	↑	Meta-analysis of case-control study [99]
Liver	+/−	↓	Cohort study [110]
Liver	+/−	↓	Meta-analysis of cohort study and case-control study [24]
Lung	↓	↑	Cohort study [111]
Lymphoma, non-Hodgikin’s	↓	+/−	Meta-analysis of cohort study and case-control study [112]
Melanoma, cutaneous	+/−	↓	Meta-analysis of cohort study [113]
Ovarian	+/−	+/−	Cohort study [103]
Prostate	+/−	+/−	Cohort study [114]
Renal cell carcinoma	+/−	+/−	Cohort study [100]
Skin cancer, non-melanoma	↓	↓	Cohort study [115]
Stomach	+/−	+/−	Meta-analysis of cohort study and case-control study [25]
Thyroid	+/−	+/−	Cohort [116]

* Risk decrease, risk increase and no effect are shown by **↓**, **↑**, and +/−, respectively.
